# Ablation versus anti-arrhythmic therapy for reducing all hospital episodes from recurrent atrial fibrillation: a prospective, randomized, multi-centre, open label trial

**DOI:** 10.1093/europace/euac253

**Published:** 2022-12-28

**Authors:** Prapa Kanagaratnam, James McCready, Muzahir Tayebjee, Ewen Shepherd, Thiagarajah Sasikaran, Derick Todd, Nicholas Johnson, Andreas Kyriacou, Sajad Hayat, Neil A Hobson, Ian Mann, Richard Balasubramaniam, Zachary Whinnett, Mark Earley, Sanjiv Petkar, Rick Veasey, Senthil Kirubakaran, Clare Coyle, Min-Young Kim, Phang Boon Lim, James O’Neill, D Wyn Davies, Nicholas S Peters, Daphne Babalis, Nicholas Linton, Emanuela Falaschetti, Mark Tanner, Jaymin Shah, Neil Poulter

**Affiliations:** National Heart and Lung Institute, Imperial College London, St Mary's Hospital, Praed Street, Paddington W2 1NY, UK; Cardiology, Hammersmith Hospital, Imperial College Healthcare NHS Trust, 72 Du Cane Rd, London, W12 0HS, UK; Department of Cardiology, Brighton & Sussex University Hospital, Brighton, UK; Department of Cardiology, Leeds Teaching Hospitals NHS Trust, Leeds, UK; Cardiology Department, Newcastle-upon-Tyne NHS Foundation Trust, Newcastle, UK; Imperial Clinical Trials Unit, School of Public Health, Imperial College London, London, UK; Cardiology, Liverpool Heart and Chest Hospital, Liverpool, UK; Imperial Clinical Trials Unit, School of Public Health, Imperial College London, London, UK; Department of Cardiology, Sheffield Teaching Hospitals NHS Trust, Sheffield, UK; Cardiology, University Hospitals Coventry & Warwickshire, Coventry, UK; Cardiology Department, Hull & East Yorkshire Hospitals NHS Trust, Hull, UK; National Heart and Lung Institute, Imperial College London, St Mary's Hospital, Praed Street, Paddington W2 1NY, UK; Cardiac Intervention Unit, Royal Bournemouth & Christchurch Hospitals NHS Trust, Bournemouth, UK; National Heart and Lung Institute, Imperial College London, St Mary's Hospital, Praed Street, Paddington W2 1NY, UK; Cardiology, Barts Health NHS Trust, London, UK; Cardiology Department, Royal Wolverhampton NHS Trust, Wolverhampton, UK; Cardiology Department, East Sussex Healthcare NHS Trust, Eastbourne, UK; Department of Cardiology, Portsmouth Hospitals NHS Trust, Portsmouth, UK; National Heart and Lung Institute, Imperial College London, St Mary's Hospital, Praed Street, Paddington W2 1NY, UK; National Heart and Lung Institute, Imperial College London, St Mary's Hospital, Praed Street, Paddington W2 1NY, UK; Cardiology, Hammersmith Hospital, Imperial College Healthcare NHS Trust, 72 Du Cane Rd, London, W12 0HS, UK; Department of Cardiology, Leeds Teaching Hospitals NHS Trust, Leeds, UK; Cardiology, Hammersmith Hospital, Imperial College Healthcare NHS Trust, 72 Du Cane Rd, London, W12 0HS, UK; National Heart and Lung Institute, Imperial College London, St Mary's Hospital, Praed Street, Paddington W2 1NY, UK; Imperial Clinical Trials Unit, School of Public Health, Imperial College London, London, UK; National Heart and Lung Institute, Imperial College London, St Mary's Hospital, Praed Street, Paddington W2 1NY, UK; Imperial Clinical Trials Unit, School of Public Health, Imperial College London, London, UK; Cardiology, Western Sussex NHS Foundation Trust, Chichester, UK; Cardiology, London North West University Healthcare NHS Trust, London, UK; Imperial Clinical Trials Unit, School of Public Health, Imperial College London, London, UK

**Keywords:** Atrial fibrillation, Ablation, Anti-arrhythmic, Radiofrequency ablation, Cryoablation

## Abstract

**Aims:**

There is rising healthcare utilization related to the increasing incidence and prevalence of atrial fibrillation (AF) worldwide. Simplifying therapy and reducing hospital episodes would be a valuable development. The efficacy of a streamlined AF ablation approach was compared to drug therapy and a conventional catheter ablation technique for symptom control in paroxysmal AF.

**Methods and results:**

We recruited 321 patients with symptomatic paroxysmal AF to a prospective randomized, multi-centre, open label trial at 13 UK hospitals. Patients were randomized 1:1:1 to cryo-balloon ablation without electrical mapping with patients discharged same day [Ablation Versus Anti-arrhythmic Therapy for Reducing All Hospital Episodes from Recurrent (AVATAR) protocol]; optimization of drug therapy; or cryo-balloon ablation with confirmation of pulmonary vein isolation and overnight hospitalization. The primary endpoint was time to any hospital episode related to treatment for atrial arrhythmia. Secondary endpoints included complications of treatment and quality-of-life measures. The hazard ratio (HR) for a primary endpoint event occurring when comparing AVATAR protocol arm to drug therapy was 0.156 (95% CI, 0.097–0.250; *P* < 0.0001 by Cox regression). Twenty-three patients (21%) recorded an endpoint event in the AVATAR arm compared to 76 patients (74%) within the drug therapy arm. Comparing AVATAR and conventional ablation arms resulted in a non-significant HR of 1.173 (95% CI, 0.639–2.154; *P* = 0.61 by Cox regression) with 23 patients (21%) and 19 patients (18%), respectively, recording primary endpoint events (*P* = 0.61 by log-rank test).

**Conclusion:**

The AVATAR protocol was superior to drug therapy for avoiding hospital episodes related to AF treatment, but conventional cryoablation was not superior to the AVATAR protocol. This could have wide-ranging implications on how demand for AF symptom control is met.

**Trial registration:**

Clinical Trials Registration: NCT02459574.

**What’s new?**

- We tested whether a streamlined approach to atrial fibrillation (AF) ablation [Ablation Versus Anti-arrhythmic Therapy for Reducing All Hospital Episodes from Recurrent (AVATAR) protocol of cryo-balloon ablation without electrical mapping and same day discharge] can achieve better symptom control in patients with paroxysmal AF compared to drug therapy
- We found the AVATAR streamlined ablation protocol was superior to drug therapy for controlling AF symptoms.
- Conventional cryoablation was not shown to be superior to the AVATAR protocol.
- This broadens the scope for delivering AF ablation by simplifying the process and so meets increasing patient demand.

## Introduction

Atrial fibrillation (AF) is the most common cardiac arrhythmia, often requiring specialist hospital care.^1^ Anti-arrhythmic drugs have been shown to be effective at controlling AF-related symptoms in placebo-controlled studies.^2,3^ Alternatively, catheter ablation to electrically isolate the pulmonary veins (PVs) can prevent AF episodes.^4^ In patients with paroxysmal AF and symptoms despite a Class I/III anti-arrhythmic agent, this PV isolation (PVI) procedure has been superior to further drug therapy at achieving freedom from ‘30 s of AF’.^5,6^ Early studies comparing PVI and drugs in patients who were naive to Class I/III anti-arrhythmic agents did not show such clear differences.^7,8^ On this background, catheter ablation is recommended after failing a Class I/III anti-arrhythmic agent but can be considered for first line treatment of symptomatic paroxysmal AF.^1^ Increasing numbers of patients are therefore seeking ablation therapy to control symptoms. Streamlining the process by reducing the resource utilization for each patient would support more patients being treated in most healthcare systems.

Technology for catheter ablation continues to evolve such that the cryo-balloon could be used without intracardiac electrical recordings.^9,10,11^ This could broaden staff and type of cardiac catheter laboratory that could deliver AF ablation. We postulated that we could reduce resource utilization further with same-day discharge and only following-up symptomatic patients. We tested these changes in a randomized, controlled trial, in which the primary objective was to determine if this streamlined approach to ablation for patients with symptomatic paroxysmal AF was superior to a drug therapy. As a secondary objective, we also tested whether standard catheter ablation practices conferred superiority over this streamlined method.

## Methods

### Study design

Ablation Versus Anti-arrhythmic Therapy for Reducing All Hospital Episodes from Recurrent Atrial Fibrillation (AVATAR-AF) was a randomized, multi-centre, open label trial testing superiority of a ‘streamlined’ AVATAR-protocol ablation procedure over drug therapy as the primary hypothesis. An AF specialist confirmed patient suitability for both arms.

A core part of clinical AF management is patient education to improve symptom control. This includes understanding that AF will self-terminate and using a ‘pill-in-the-pocket’ if needed. If done properly, the majority of patients with paroxysmal AF will avoid the Emergency Room and hospital admission. However, these episodes will still be debilitating; therefore, it is essential that hospital outpatient episodes are included to capture all patients who continue to suffer significant symptoms from paroxysmal AF. Therefore, we used a primary outcome measure that included outpatient hospital episodes, for patients who are symptomatic and need modification of their AF treatment strategy, as well as those needing emergency care.

Confirming PVI is considered essential to the AF ablation procedure. We addressed this as a secondary hypothesis using a second control arm of cryo-balloon AF ablation following standard practice and testing for superiority over the ‘streamlined’ AVATAR approach.

The trial was sponsored by Imperial College London (London, UK) and approved by the Health Research Authority [London - Brent Local Research Ethics Committee (14/LO/0117)]. The Trial Steering Committee was responsible for study conduct, oversight, and reporting. The study was adopted onto the National Institute of Health Research Portfolio and received support from the Comprehensive Local Research Network which provided research nurses at each site. An independent Data Safety Monitoring Board ensured optimal therapy was delivered according to protocol. An Endpoints Adjudication Committee was blinded to assigned treatment and used specified definitions to validate all reported endpoints. Trial management, quality control, quality assurance, data management, and statistical analysis were provided by the United Kingdom clinical research collaboration (UKCRC)-registered Imperial Clinical Trials Unit. The rationale and design of the AVATAR-AF study has been published.^12^

### Patients

Patients with documented symptomatic paroxysmal AF were recruited from UK hospitals. They had to be suitable for either modification of drug therapy or catheter ablation. Full eligibility criteria are presented in the trial design manuscript and Protocol (see Supplementary Materials).^12^ All participants provided written informed consent. Patients or the public were not involved in the design, conduct, or reporting plans for the research, but we plan to disseminate the results to relevant patient organizations.

### Procedures

Randomization was assigned in a 1:1:1 ratio, stratified by site in block sizes of six, to either streamlined AVATAR-protocol ablation, optimized anti-arrhythmic therapy, or conventional AF cryo-balloon ablation.

Interventions were applied at fixed time points during a 12-week period with the aim of discharging the patient from specialist hospital-based treatment.

In the AVATAR-protocol ablation group, the Arctic Front Advance™ cryoballoon (Medtronic, Minneapolis, MN, USA) was used to occlude PVs to achieve minimal/no dye leak on injection. No intra-cardiac electrical recording catheters were deployed, and PVI was not formally assessed. A bedside transthoracic echocardiogram, haemoglobin check, and femoral puncture site assessment were carried out 6 h post-procedure. If all parameters were stable, the patient was discharged on the day of intervention. This was included in the randomized part of the protocol as it has not been tested previously in a controlled clinical trial. Therefore, we did not define it as standard practice even though it is becoming more common.

In the conventional AF ablation group, cryo-ablation efficacy was monitored with a circular mapping catheter of the operator’s choice. PVI was completed with either repeat cryo-balloon application, deflectable cryotherapy catheter, or radiofrequency catheter as per operator preference. Post-procedural tests were the same as the AVATAR arm but discharged the next day.

During the 12-week treatment period, all pre-ablation anti-arrhythmic agents were continued for 4 weeks post-ablation and then reduced with the aim of stopping. Repeat ablations are required for patients with symptoms due to recurrent AF from PV reconnection. Therefore, a second ablation was permitted at 10 weeks for recurrent AF symptoms as a part of the initial ablative treatment strategy.

In the drug therapy arm, patients were reviewed every 4 weeks to optimize anti-arrhythmics. Drug choice and dose recommendation were in accordance with local and international guidelines. At each review, the medication could be left unchanged, the dose altered, or changed to a different agent as required. Drug dosing was optimized to achieve symptom control without side effects. In patients with frequent symptoms, this timescale was expected to be sufficient to maximize drug dosages and in those who had infrequent symptoms to achieve lower maintenance doses with a ‘pill-in-the-pocket’ plan for breakthrough episodes.

Patients who were symptomatically improving at the 12-week review were discharged from hospital-based specialist arrhythmia care. Patients who had ongoing symptoms or problems related to their assigned therapy were deemed to have ‘failed 12-week discharge’ and counted as a primary endpoint. Telephone interviews every 3 months ensured appropriate primary care engagement. Hospital-based attendance, investigations, and treatments were adjudicated by a blinded Endpoints Committee.

### Outcomes

The primary endpoint was time to any hospital episode related to treatment for atrial arrhythmia following the 12-week treatment period. We used the term ‘hospital episode’ to include inpatient stay, attendance to an emergency room, or outpatient clinic to be reviewed by an arrhythmia specialist. This reflected the entire healthcare utilization of a patient whose AF was not well controlled. This endpoint would not capture all AF episodes occurring in the patient but transforms the subjectivity of symptomatic recurrences to measurable events requiring healthcare resources.

The secondary endpoints were time to death or stroke from any cause, complications caused by procedure or anti-arrhythmic drug, and all hospital episodes resulting in a change in therapy for atrial arrhythmia.

Quality-of-life questionnaires [atrial fibrillation Quality of life survey (AFQT) and EQ-5D-5L] were completed at randomization and 1 year.

### Statistical analysis

In this time-to-event study of hospital episodes, the sample size was estimated using the log-rank test under the Freedman method based on the occurrence of first event during the 12-month follow-up period. The null hypothesis was that there was no difference in the distributions. An initial two-sided alpha of 0.05 was chosen, and given the need to allow for the inherent multiple testing between the three arms of the study, the conservative Bonferroni correction was applied and the alpha value used in the power calculations was reduced to 0.025.

Estimating the proportion experiencing a hospital episode in the follow-up period in the anti-arrhythmic arm as 0.63 and the corresponding proportion in the AVATAR ablation arm as 0.40, assuming the occurrence of 103 events, with 100 evaluable patients in each of the two treatment arms yields a power of 0.80. A similar power is obtained for the comparison of the AVATAR ablation arm (0.40) and the conventional AF ablation arm (0.20). These estimates were based on published data for ‘freedom from 30 s of AF’. Using a loss to follow-up of 7%, a recruitment target of 321 patients was set.

Treatment was considered failed when a patient experienced any hospital episode related to treatment for atrial arrhythmia. Time to failure was the time from first intervention to time of event. The ‘decision not to discharge’ was used as the endpoint for analysis in those patients who were not discharged after the 12-week treatment period.

Data were analysed using intention-to-treat principle, and a *P*-value of 0.025 or less considered significant. Primary outcome was analysed using Kaplan–Meier statistics. Differences between treatment arms were assessed using log-rank testing and proportional hazards models. A sensitivity per-protocol analysis was performed, removing patients who failed to receive their allocated intervention or underwent cross-over to an alternative treatment before the week 12 discharge review. Secondary endpoints of time to death or stroke; time to procedural/drug-based complication were analysed using Kaplan-Meier and descriptive statistics. Due to the low frequency of secondary endpoint events, the results are presented as frequency of events and differences assessed using Fisher’s Exact test. In addition to the primary analysis above, the model was adjusted to account for the following baseline co-variates: site, weight, hypertension, number of anti-arrhythmics taken by patient prior to first intervention.

## Results

**Figure 1:**
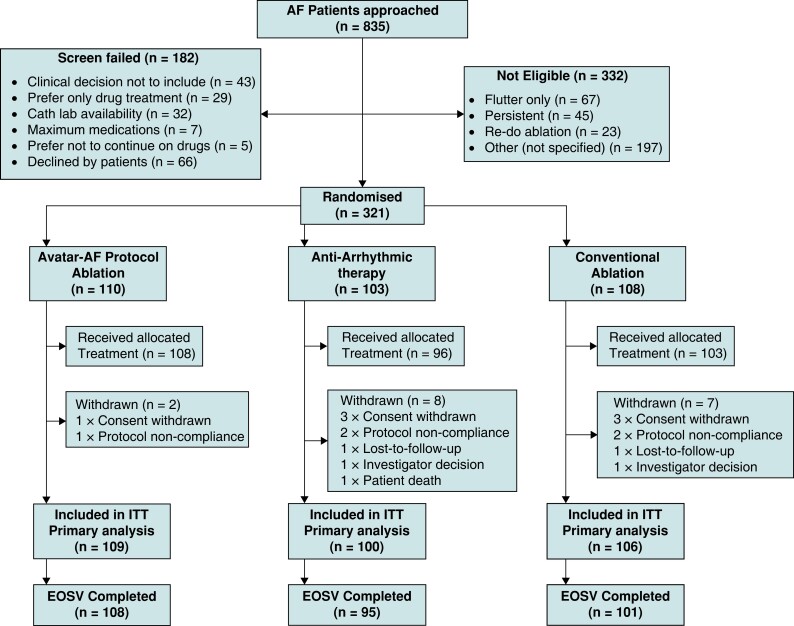
Screening, randomization, treatment, and follow-up. Consort diagram showing the patient numbers at each stage of the study. Patients referred to an arrhythmia specialist with a diagnosis of paroxysmal atrial fibrillation (AF) were screened. Enrolment was offered to those considered clinically suitable for any of the three treatment arms. Randomization took place once the treatment schedule was confirmed to be feasible irrespective of the assigned arm.

From April 2015 to August 2017, 835 patients were approached; 182 failed screening, 332 were not eligible (*Figure 1*), and 321 patients were recruited and randomized. One hundred ten patients were assigned to the AVATAR protocol arm, 103 patients to the anti-arrhythmic arm, and 108 patients to the conventional ablation arm. Patient characteristics are shown in *Table 1*.

**Table 1:** Baseline characteristics

Characteristic		AVATAR protocol arm	Anti-arrhythmic arm	Conventional ablation arm
		(*n* = 110)	(*n* = 103)	(*n* = 108)
Age	Mean (SD)	60.1 (9.00)	60.5 (10.34)	59.7 (12.22)
Male sex	Number (%)	64 (58)	57 (55)	66 (61)
Body mass index	Mean (SD)	29.1 (5.04)	28.0 (4.83)	28.9 (4.67)
Hypertension	Number (%)	38 (35)	36 (35)	29 (27)
Diabetes mellitus	Number (%)	4 (4)	6 (6)	4 (4)
Sleep apnoea	Number (%)	1 (1)	3 (3)	0
Coronary heart disease	Number (%)	3 (3)	3 (3)	4 (4)
CHADSVASC score	0	35 (32)	36 (35)	43 (40)
	1	30 (27)	24 (23)	22 (20)
	2	28 (26)	19 (18)	23 (21)
	3	12 (11)	17 (14)	10 (9)
	4	4 (4)	4 (4)	6 (6)
	5	0	1 (1)	1 (1)
	6	0	1 (1)	2 (2)
	Not known	1 (1)	1 (1)	1 (1)
Left ventricular function (ejection fraction)	Mean (SD)	57.1 (4.88)	57.9 (5.60)	58.3 (5.00)
Anti-coagulation at enrolment	All agents (%)	81 (74)	65 (63)	76 (70)
	Warfarin	13 (12)	14 (14)	22 (20)
	Apixaban	30 (27)	23 (22)	26 (24)
	Rivaroxaban	30 (27)	22 (21)	21 (19)
	Dabigatran	6 (6)	5 (5)	4 (4)
	Edoxaban	2 (2)	1 (1)	3 (3)

AVATAR, Ablation Versus Anti-arrhythmic Therapy for Reducing All Hospital Episodes from Recurrent.

In the AVATAR arm, 108 of 110 patients (98%) underwent the planned ablation. Of these, 92 (84% of patients randomized) were discharged the same day. Seventeen patients (16%) were referred for a redo procedure at the 8-week review of whom 13 (12%) completed the procedure. Ninety-four patients (85%) achieved symptom control and were discharged from specialist hospital care following review at 12 weeks. Protocol deviations and peri-procedural characteristics are shown in Supplementary material online, *Appendix Tables S1* and *S2*.

In the anti-arrhythmic arm, 96 patients (93%) attended the initial review. The 4-week review was attended by 89 patients (86%) and the 8-week review by 91 patients (88%). At each review, anti-arrhythmic treatment was optimized, and 93 patients (90%) had a change in medication. At 12 weeks, 47 patients (46%) had achieved symptom control and were discharged from specialist hospital care, and 2 patients had crossed over and completed ablation.

In the conventional ablation arm, 103 of 108 underwent the planned ablation (95%). Ninety-nine patients had confirmed PVI (92%). Ten patients (10%) were referred for a redo procedure at 8-week review, of whom 7 (7%) completed the redo procedure. Ninety-seven patients (90%) were discharged from specialist hospital care following review at 12 weeks.

Of those who attended the 12-week review, 78 patients (76%) in the drug therapy arm were taking a Class I/Class III anti-arrhythmic agent compared to 11 (11%) in the AVATAR arm and 12 (12%) in the conventional ablation arm (*Table 2* details the agents and changes, Supplementary material online, *Appendix Table S3* the final doses). There was equivalent ongoing use of the ‘pill-in-the-pocket’ strategy in all three arms of about 8%.

**Table 2:** Drug changes during 12week treatment pathway

		AVATAR arm		Anti-arrhythmic arm		Conventional arm	
		At randomization (*n* = 110)	At week 12 discharge (*n* = 105)	At randomization (*n* = 103)	At week 12 discharge (*n* = 92)	At randomization (*n* = 108)	At week 12 discharge (*n* = 103)
Number of agents used for symptom control	None, *n* (%)	24 (21.8)	58 (55.2)	20 (19.4)	7 (7.6)	18 (16.7)	57 (55.3)
	Single agent, *n* (%)	68 (61.8)	40 (38.1)	71 (68.9)	49 (53.3)	73 (67.6)	39 (37.8)
	Two agents, *n* (%)	18 (16.4)	6 (5.7)	12 (11.7)	36 (39.1)	17 (15.7)	7 (6.8)
	Three agents, *n*(%)	—	1 (1.0)	—	—	—	—
Number of Class I/III agents	None, *n* (%)	68 (61.8)	94 (89.5)	65 (63.1)	18 (19.6)	69 (63.9)	91 (88.3)
	Single agent, *n* (%)	42 (38.2)	11 (10.5)	38 (36.9)	74 (80.4)	39 (36.1)	12 (11.8)
All beta-blockers, *n* (%)		61 (55.4)	37 (35.2)	54 (52.4)	44 (47.8)	65 (60.1)	35 (34.0)
All calcium channel blockers *n* (%)		8 (7.3)	7 (6.6)	7 (6.8)	6 (6.5)	9 (8.3)	6 (5.8)
All Class I/III anti-arrhythmics^a^, *n* (%)		34 (30.9)	11 (10.5)	34 (33.0)	70 (76.1)	32 (29.6)	12 (11.7)

Beta blockers^a^	Patients on drug at start, *n* (%)	Change in total daily drug dose for cohort	Patients on drug at 12 weeks, *n*(%)	Patients on drug at start, *n,*(%)	Change in total daily drug dose for cohort	Patients on drug at 12 weeks, *n* (%)	Patients on drug at start, *n* (%)	Change in total daily drug dose for cohort	Patients on drug at 12 weeks, *n* (%)
Atenolol	2 (1.8)	−43	1 (1.0)	3 (2.9)	−34	2 (2.2)	1 (0.9)	+2	1 (1.0)
Bisoprolol	57 (51.8)	−160	36 (34.3)	47 (45.6)	−12	40 (43.5)	62 (57.4)	−95	34 (33.0)
Metoprolol	0	0	0	3 (2.9)	−134	1 (1.1)	1 (0.9)	−139	0
Nebivolol	2 (1.8)	−4	0	1 (1.0)	0	1 (1.1)	1 (0.9)	−1	0
Calcium channel blockers^a^	Patients on drug at start, *n* (%)	Change in total daily drug dose for cohort	Patients on drug at 12 weeks, *n* (%)	Patients on drug at start, *n* (%)	Change in total daily drug dose for cohort	Patients on drug at 12 weeks, *n* (%)	Patients on drug at start, *n* (%)	Change in total daily drug dose for cohort	Patients on drug at 12 weeks, *n* (%)
Diltiazem	5 (4.5)	+434	6 (5.7)	5 (4.9)	+314	6 (6.5)	7 (6.5)	−61	6 (5.8)
Verapamil	3 (2.7)	−540	1 (1.0)	2 (1.9)	−466	0	2 (1.9)	−444	0
Class I/III anti-arrhythmics^a^	Patients on drug at start, *n* (%)	Change in total daily drug dose for cohort	Patients on drug at 12 weeks, *n* (%)	Patients on drug at start, *n* (%)	Change in total daily drug dose for cohort	Patients on drug at 12 weeks, *n* (%)	Patients on drug at start, *n* (%)	Change in total daily drug dose for cohort	Patients on drug at 12 weeks, *n* (%)
Amiodarone	4 (3.6)	−636	0	1 (1.0)	+1110	4 (4.3)	2 (1.9)	−185	0
Dronedarone	1 (0.9)	−727	0	1 (1.0)	+5980	8 (8.7)	1 (0.9)	−741	0
Flecainide	21 (19.1)	−1913	7 (6.7)	21 (20.4)	+3856	38 (41.3)	18 (16.7)	−1289	9 (8.7)
Propafenone	1 (0.9)	+578	2 (1.9)	1 (1.0)	+867	3 (3.3)	1 (0.9)	−556	0
Sotalol	7 (6.4)	−611	2 (1.9)	10 (9.7)	+1327	17 (18.5)	10 (9.3)	−863	3 (2.9)

AVATAR, Ablation Versus Anti-arrhythmic Therapy for Reducing All Hospital Episodes from Recurrent.

Drugs taken as pill-in-the-pocket are not included as regular medications.

### Primary endpoints

**Figure 2:**
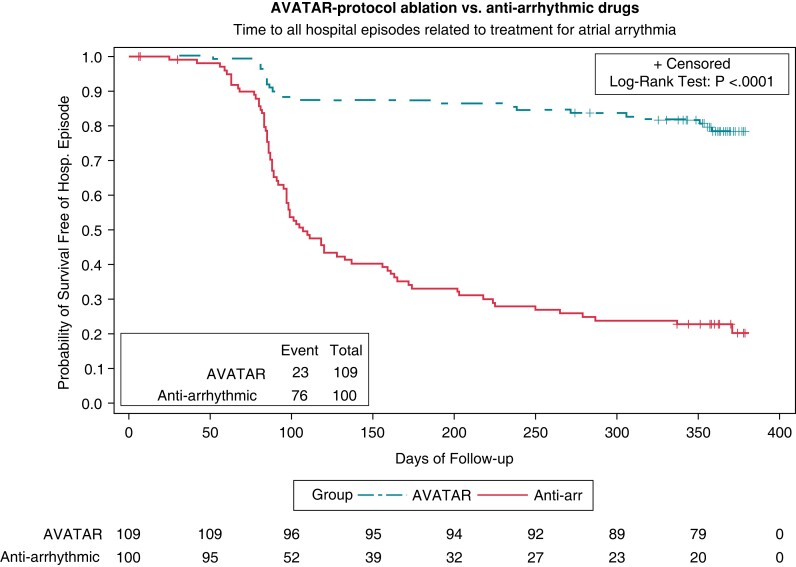
Ablation Versus Anti-arrhythmic Therapy for Reducing All Hospital Episodes from Recurrent (AVATAR) protocol ablation vs. anti-arrhythmic drug therapy: primary endpoint analysis. Kaplan–Meier curves comparing AVATAR protocol ablation against drug therapy for the survival time to any hospital episode related to treatment for atrial arrhythmia. Number of remaining subjects at risk displayed at 50-day intervals. The hazard-ratio for achieving a hospital episode when comparing AVATAR protocol arm to drug therapy is 0.156 (95% CI, 0.097–0.250; *P* < 0.0001 by Cox regression). Comparing the survival distributions between the two groups, significantly fewer patients had reached the primary endpoint with 23 (21%) vs. 76 (74%) patients having events within the two arms, respectively (*P* < 0.0001 by log-rank test).

**Figure 3:**
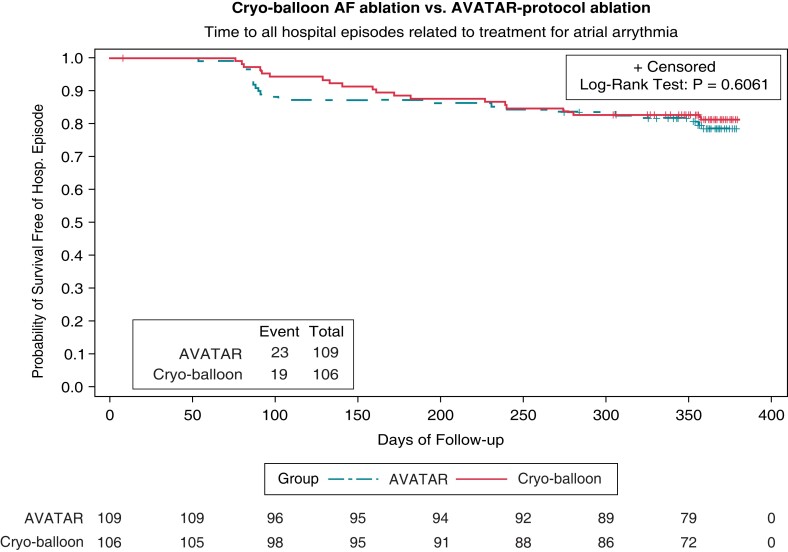
Cryo-balloon atrial fibrillation (AF) ablation vs. Ablation Versus Anti-arrhythmic Therapy for Reducing All Hospital Episodes from Recurrent (AVATAR) protocol ablation: primary endpoint analysis. Kaplan–Meier curves comparing AVATAR protocol ablation against cryo-balloon AF ablation therapy for the survival time to any hospital episode related to treatment for atrial arrhythmia. Number of remaining subjects at risk displayed at 50-day intervals. The hazard ratio for achieving a hospital episode when comparing the AVATAR protocol arm against the conventional cryo-balloon ablation arm is HR 1.173 (95% CI, 0.639 to 2.154; *P* = 0.61 by Cox regression). No significant difference was found when comparing the survival distribution between the two groups with 23 (21%) vs. 19 (18%) patients having events within the two arms respectively (*P* = 0.61 by log-rank test).

The hazard ratio (HR) for achieving a hospital episode when comparing AVATAR protocol arm to drug therapy is 0.156 (95% CI, 0.097–0.250; *P* < 0.0001 by Cox regression). Significantly fewer patients had reached the primary endpoint with 23 (21%) vs. 76 (74%) patients having events within the two arms, respectively (*P* < 0.0001 by log-rank test). The Kaplan–Meier curves for the primary endpoint of the primary hypothesis are shown in *Figure 2*. There was no significant HR when comparing the AVATAR protocol arm against the conventional cryo-balloon ablation arm (HR 1.173, 95% CI, 0.639–2.154; *P* = 0.61 by Cox regression), with endpoint frequencies of 23 (21%) vs. 19 (18%) (*P* = 0.61 by log-rank test). The Kaplan–Meier curves for the primary endpoint of the secondary hypothesis are shown in *Figure 3*. Post-hoc testing exploring absolute risk indicates that the estimated proportion of those achieving endpoint within the AVATAR protocol arm is 0.209 (95% CI, 0.113–0.285) and in the cryo-balloon ablation arm 0.176 (0.104–0.248). The corresponding difference in absolute risk is 0.032 (−0.071 to 0.138). Results were unchanged throughout the per-protocol sensitivity analysis and when adjusting the proportional hazards model for baseline covariates. A breakdown of the adjudicated primary endpoints is shown in *Table 3*.

**Table 3:** Breakdown of endpoints

Endpoint description	AVATAR arm		Anti-arrhythmic arm		Conventional arm	
	*n* = 110		*n* = 103		*n* = 108	
Primary endpoints	23 (20.9%)		76 (73.8%)		19 (17.6%)	
Primary endpoint timing	Failed 12-week discharge	After 12 weeks	Failed 12-week discharge	After 12 weeks	Failed 12-week discharge	After 12 weeks
	9 (8.2%)	14 (12.7%)	36 (35.0%)	40 (38.8%)	6 (5.6%)	13 (12.0%)
ER visit leading to inpatient admission	1 (0.9%)		3 (2.9%)		4 (3.7%)	
ER visit leading to discharge	2 (1.8%)		4 (3.9%)		1 (0.9%)	
Outpatient consultation	20 (18.2%)		69 (67.0%)		14 (13.0%)	
Symptoms of atrial arrhythmia with ECG documentation at primary endpoint	16 (14.5%)		25 (24.3%)		9 (8.3%)	
Symptoms of atrial arrhythmia without ECG documentation at primary endpoint	7 (6.4%)		43 (41.7%)		10 (9.3%)	
Treatment complication/drug side effects at primary endpoint	0		8 (7.8%)		0	
Ablation after primary endpoint	9 (8.2%)		53 (51.5%)		15 (13.9%)	
Medication alteration after primary endpoint	4 (3.6%)		6 (5.8%)		2 (1.9%)	
Investigations only after primary endpoint	7 (6.4%)		16 (15.5%)		1 (0.9%)	
No action after primary endpoint	3 (2.7%)		1 (1.0%)		1 (0.9%)	

AVATAR, Ablation Versus Anti-arrhythmic Therapy for Reducing All Hospital Episodes from Recurrent.

### Secondary endpoints

Complications related to treatment are shown in Supplementary material online, *Appendix Table S4*. One death occurred in the anti-arrhythmic arm shortly after starting sotalol. Two transient ischaemic attacks occurred; one each in anti-arrhythmic and conventional ablation arms. Eighteen patients in the anti-arrhythmic arm had side effects related to drug therapy.

Quality-of-life was measured using the AFQT and EQ-5D-5L questionnaires at randomization and 1 year. All arms demonstrated a significant improvement in quality of life over 1 year (see Supplementary material online, *Appendix Table S5A* and *S5B*). There was no difference between the AVATAR-AF arm and the drug therapy or conventional ablation arms. However, the drug therapy arm included patients who had received ablation therapy after the primary endpoint.

### Ablation after index intervention

Patients were allowed a redo-ablation during the 12-week treatment schedule without triggering an endpoint (see Supplementary material online, *Appendix Figure S1*). Nine patients (8%) in the AVATAR arm, and six patients (6%) in the conventional ablation arm entered the follow-up phase with a redo-ablation. During the follow-up period, 9 patients (8%) in the AVATAR arm had a redo procedure following a primary endpoint compared to 15 patients (15%) in the conventional ablation arm. In the drug therapy arm, 56 of 103 patients (54%) went on to have an ablation during the follow-up period. The total redo-ablation rate (within 12 weeks and after endpoint) in the AVATAR arm was 22 patients (20%) compared to 22 patients (22%) in the conventional ablation arm. Therefore, in terms of resource utilization, the redo rates in the two ablation arms were not different.

### Ablation as ‘first-line’ therapy

Post-hoc analysis of ablation therapy as ‘first-line therapy’ prior to initiation of Class I/III anti-arrhythmic agents showed similar outcomes. The AVATAR arm had significantly fewer primary endpoints [14 patients (21% of 67 Class I/III naïve patients) vs. 44 patients (70% of 63 Class I/III naïve patients), *P* < 0.0001 by log rank rest)] than patients in the drug therapy arm (see Supplementary material online, *Appendix Figure S2*). Therefore, the AVATAR protocol was also superior to drug therapy as ‘first-line’ treatment.

### Procedural parameters

More patients had two cryo-applications in all veins in the AVATAR group (as per protocol) compared to the conventional ablation (see Supplementary material online, *Appendix Table S6*). PV mapping in the conventional arm would have shown that the first freeze had already electrically isolated the vein so the operator chose not to deliver the second. Amongst all patients having ablation, the use of two applications was associated with fewer endpoints (see Supplementary material online, *Appendix Table S7*).

## Discussion

The AVATAR-AF trial has shown that a streamlined approach to AF ablation was superior to drug therapy at avoiding hospital-based treatment for AF. This was primarily driven by recurrent symptoms leading to outpatient consultations. The standard catheter ablation approach did not demonstrate superior outcomes to the streamlined AVATAR approach. All three arms had significant improvement in quality-of-life measures over the year, but there was no difference between the AVATAR arm and the two control groups.

AVATAR-AF reports the outcomes from AF treatments on the basis of being able to avoid hospital episodes including consultations and admissions. We believe this provides a measure of symptom burden that is easy for patients to understand and is a relevant metric to healthcare providers. Similarly, the recent Early Treatment of Atrial Fibrillation for Stroke Prevention Trial 4 (EAST-AFNET 4) showed benefit using a clinically unambiguous composite endpoint to confirm that trying to maintain sinus rhythm using drugs, cardioversion and ablation was superior to rate control in AF.^13^ However, when comparing catheter ablation with medical therapy in the catheter ablation vs antiarrhythmic drug therapy for atrial fibrillation (CABANA) trial, it was not possible to show a difference in a similar composite endpoint. This was despite catheter ablation achieving a significant reduction in AF recurrences documented by ECG recordings.^14^ ECG documentation of ‘30 s of AF’ has been used extensively as an endpoint in catheter ablation trials to show superiority over drug therapy.^4,5,6^ Even the most recent studies such as Early Aggressive Invasive Intervention for Atrial Fibrillation (EARLY-AF) and Cryoballoon Catheter Ablation in an Antiarrhythmic Drug Naive Paroxysmal Atrial Fibrillation (STOP-AF-FIRST) included ‘recurrence of 30 s of AF’ within their primary endpoint.^15,16^ This endpoint includes asymptomatic patients whose quality of life should not be affected by this type of ‘treatment failure’ and, as demonstrated by the CABANA trial, does not appear to correlate with clinically important composite endpoints.^17,18^ Unlike these studies, AVATAR-AF used a primary endpoint to account for symptomatic recurrences which affect patient and physician perception of treatment efficacy. The results provide direct validation of guideline recommendations for catheter ablation, proving symptom benefit for a symptom-based indication.^1^ In addition, post-hoc analysis shows that amongst patients naive to Class I/III anti-arrhythmic agents, the AVATAR approach led to fewer hospital episodes (inpatient and outpatient) than drug therapy. This supports the findings of the EARLY-AF and STOP-AF-FIRST trials and the most recent ESC guidelines which have upgraded support for AF ablation as a first line therapy.^1,15,16^

AVATAR-AF is also the first randomized trial to simplify ablation therapy. The cryo-balloon is unique in not requiring intracardiac electrical recordings to deliver therapy. It is manipulated around the PV ostium until occlusion of PV flow can be demonstrated using a contrast injection under fluoroscopy. Cryotherapy delivered during complete PV occlusion is associated with a very high rate of PV electrical isolation enabling the streamlined ablation protocol.^10,11^ However, not checking for PVI could result in ‘incomplete’ ablation and a higher recurrence rate, but the overall redo-ablation rates following the index ablation were similar in the two ablation arms over the entire study period. The majority of patients having PVI procedures will have electrical reconnection of the PVs by 3 months,^19^ but only a proportion will be associated with AF recurrence and need a redo-ablation procedure. Our study suggests that for the cryo-balloon, PV mapping did not reduce the long-term rates of redo-ablation for symptomatic AF.

In addition, we analysed the procedural parameters post-hoc and more patients had two cryo-applications in all veins in the AVATAR group (as per protocol) compared to the conventional ablation. Amongst all patients having ablation, the use of two applications was associated with fewer endpoints. This suggests that receiving two cryo-applications per vein was more important than proving electrical isolation. This raises important questions about how best to achieve durable PVI.

The main limitation of our study design was that we assumed the prevailing view that ablation with PV mapping would be superior to the AVATAR approach. A non-inferiority study would be required to determine if the two approaches are indeed equivalent.

AVATAR-AF demonstrates that the ablation procedure can be simplified and streamlined and still be superior to medical therapy using practical patient-based outcomes across a broad range of patient groups. This potentially allows the AVATAR approach to be delivered in cardiac catheter labs without specialist electrophysiology equipment and associated expert staff. Similarly, the operator would have to be expert in accessing and manipulating catheters in the left atrium, as well as managing complications, but would not need to be able to interpret intracardiac electrograms. This ‘streamlining’ would increase the capacity for delivering AF ablation, even though we did not expect the AVATAR protocol to have an impact on procedure times. Reducing this resource requirement has broad implications for where and how the treatment is delivered and could promote more widespread provision of AF ablation. We did not test this directly in AVATAR-AF, but we have the clinical justification to proceed to such studies, especially given the increasing demand for AF therapy.^20^

We conclude that patients with symptomatic paroxysmal AF can be treated effectively by a streamlined ablation approach using a cryo-balloon.

## Transparency declaration

The lead author affirms that this manuscript is an honest, accurate, and transparent account of the study being reported; that no important aspects of the study have been omitted; and that any discrepancies from the study as planned (and registered) have been explained. All authors had full access to all of the data. PK, NJ, TS, EF, DB, NP take responsibility for the integrity of the data and accuracy of the data analysis.

## Dissemination plan

Trial results will be released in several manuscripts providing outcomes of the trial as a whole. All publications and presentations relating to the trial will be authorized by the Trial Steering Committee.

## Authors’ contributions

P.K. was the lead for conceptualization and writing-original draft and contributed to data curation, formal analysis, funding acquisition, investigation, methodology, project administration, validation. J.M.C., M.T., E.S., and D.T. contributed to data curation, formal analysis, funding acquisition, investigation, methodology, project administration, validation, and writing-review and editing. T.S., N.J., D.B., E.F., and N.P. contributed to data curation, formal analysis, project administration, validation, and writing-review and editing. I.M., C.C., and M.-Y.K. contributed to formal analysis, project administration, validation, and writing-review and editing. A.K., S.H., N.A.H., R.B., Z.W., M.E., S.P., R.V., S.K., P.B.L., J.O.N., D.W.D., N.S.P., N.L., M.T., and J.S. contributed to project administration, validation, and writing-review and editing.

## Supplementary Material

euac253_Supplementary_Data

## Data Availability

The data collected in the study, including anonymized individual patient data and a data dictionary defining each field in the data set, will be made available to others on reasonable request to the corresponding author.
