# Supplementary material for: Ablation versus anti-arrhythmic therapy for reducing all hospital episodes from recurrent atrial fibrillation: a prospective, randomized, multi-centre, open label trial
Source: Europace. 2022 Dec 28;25(3):863–72. doi: 10.1093/europace/euac253 (PMC10062288; doi:10.1093/europace/euac253)
Supplement: euac253_Supplementary_Data [file euac253_supplementary_data.docx]

**SUPPLEMENTARY MATERIALS:**

**APPENDIX TABLE 1: Summary of Protocol Deviations**

| **Type of Protocol Deviation** | **Numbers** |
| --- | --- |
| Didn't receive assigned treatment | 5 |
| Crossover arm before W12 | 5 |
| Study Investigations not performed | 22 |
| QOL questionnaire deviation | 16 |
| All Procedural deviation | 14 |
| All Drug deviations | 7 |
| All visits/telephone follow-up timeline deviations | 187 |
| Others | 2 |

**APPENDIX TABLE 2: Peri-procedural Data**


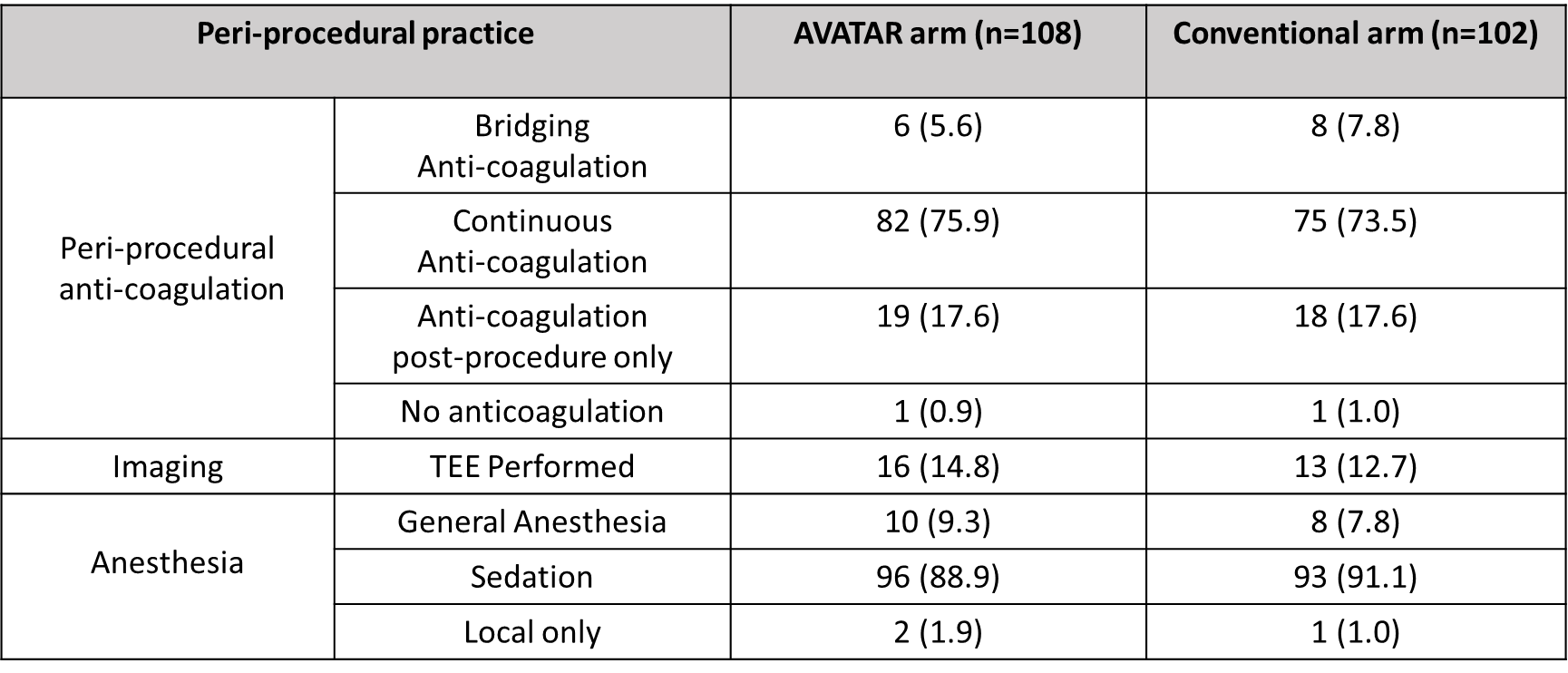


**APPENDIX TABLE 3: Drug Doses in Anti-Arrhythmic Arm**

| **Drug** | **Dose (mg)** | **Frequency** | **Daily dose (mg)** | **Number of Patients in Anti-Arrhythmic Arm taking specified dose at 12 weeks** |
| --- | --- | --- | --- | --- |
| Patients started on de novo Class I/III antiarrhythmics remaining on them (n=43) | | | | |
| Flecainide | 50 | OD | 50 | 1 (2%) |
|  | 50 | BD | 100 | 12 (28%) |
|  | 75 | BD | 150 | 2 (5%) |
|  | 100 | BD | 200 | 7 (16%) |
|  | 150 | BD | 300 | 3 (7%) |
| Sotalol | 40 | BD | 80 | 5 (12%) |
|  | 80 | OD | 80 | 1 (2%) |
|  | 80 | BD | 160 | 4 (9%) |
| Propafenone | 150 | BD | 300 | 1 (2%) |
| Amiodarone | 200 | OD | 200 | 2 (5%) |
| Dronedarone | 400 | BD | 800 | 5 (12%) |
| Patients on Class I/III antiarrhythmics at randomisation and remaining on them (n=31) | | | | |
| Flecainide | 50 | OD | 50 | 1 (3%) |
|  | 50 | BD | 100 | 6 (19%) |
|  | 100 | BD | 200 | 4 (13%) |
|  | 150 | BD | 300 | 2 (6%) |
|  | 50mg | PRN | PRN | 2 (6%) |
|  | 100mg | PRN | PRN | 2 (6%) |
| Sotalol | 40 | BD | 80 | 1 (3%) |
|  | 80 | BD | 160 | 3 (10%) |
|  | 120 | BD | 240 | 3 (10%) |
| Propafenone | 150 | TDS | 450 | 2 (6%) |
| Amiodarone | 200 | OD | 200 | 2 (6%) |
| Dronedarone | 400 | BD | 800 | 3 (10%) |
| Drugs for symptom control in patients not taking Class I/III agents at 12 weeks (n=18) | | | | |
| Bisoprolol | 1.25 | OD | 1.25 | 2 (7%) |
|  | 2.5 | OD | 2.5 | 2 (7%) |
|  | 2.5 | BD | 5 | 1 (3%) |
|  | 5 | OD | 5 | 1 (3%) |
|  | 5 | BD | 10 | 1 (3%) |
| Diltiazem | 120 | OD | 120 | 1 (3%) |
|  | 120 | BD | 240 | 1 (3%) |
| Atenolol | 25 | OD | 25 | 1 (3%) |
|  | 100 | OD | 100 | 1 (3%) |
| No medication for AF symptom control | | | | 7 (24%) |

**APPENDIX TABLE 4: Complications during 1 year follow-up period**

**APPENDIX TABLE 5A: Quality of Life Outcomes (AFEQT)**

**APPENDIX TABLE 5B: Quality of Life Outcomes (EQ-5D-5L)**

**APPENDIX TABLE 6: Procedural Comparison between AVATAR and Conventional arms**

**
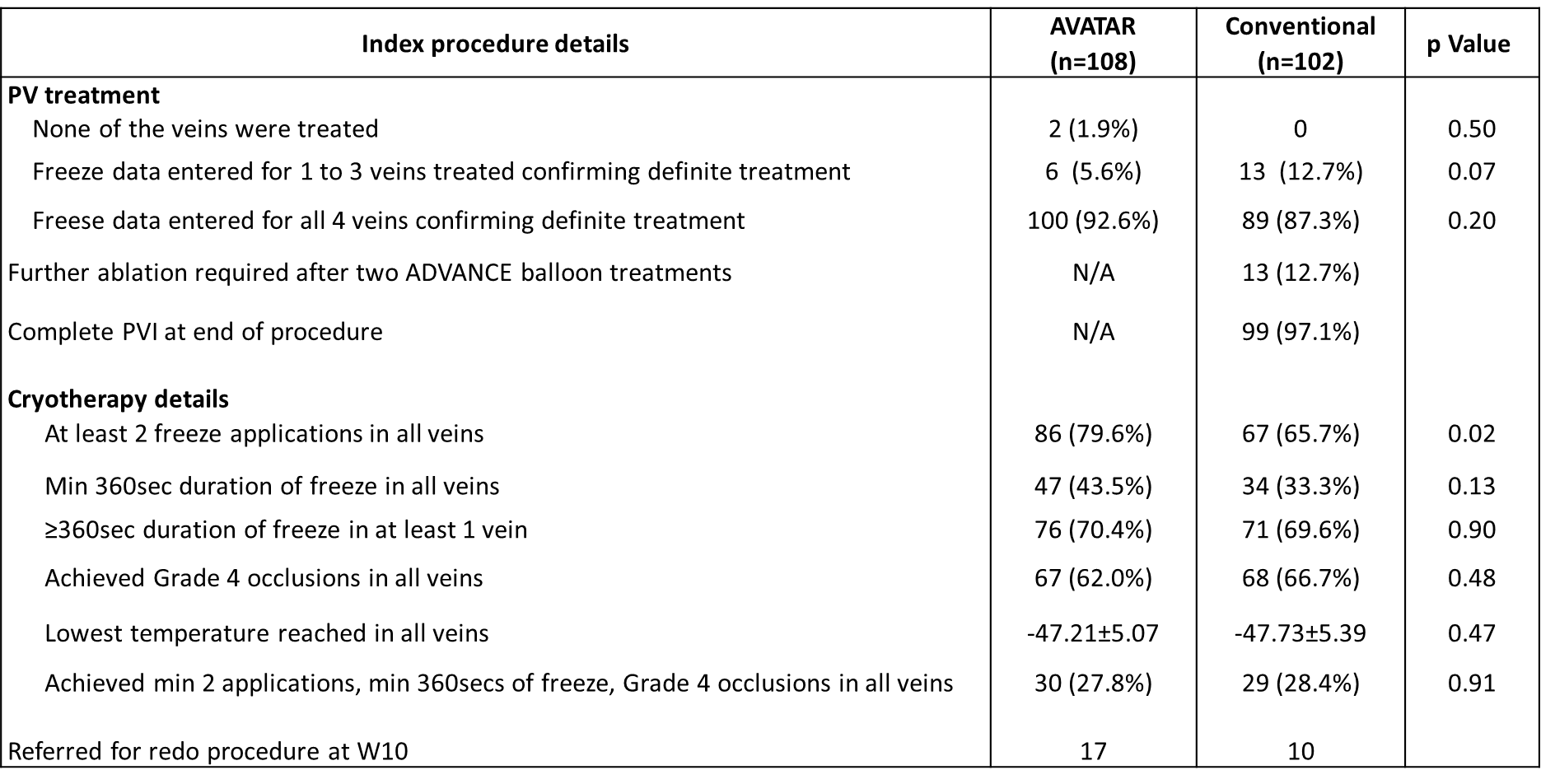
**

**APPENDIX TABLE 7: Procedural Comparison between ablation patients reaching primary endpoint**

**
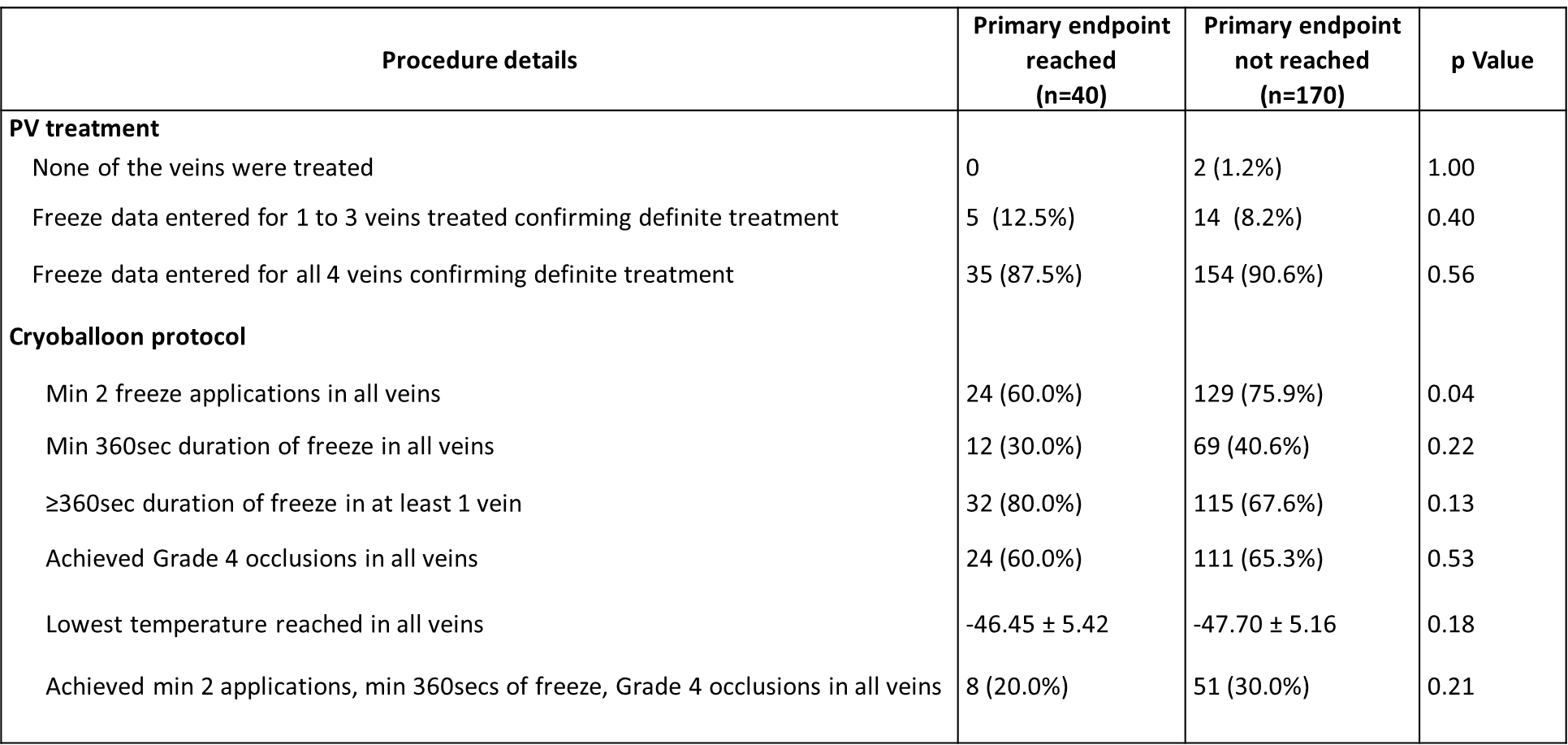
**

**
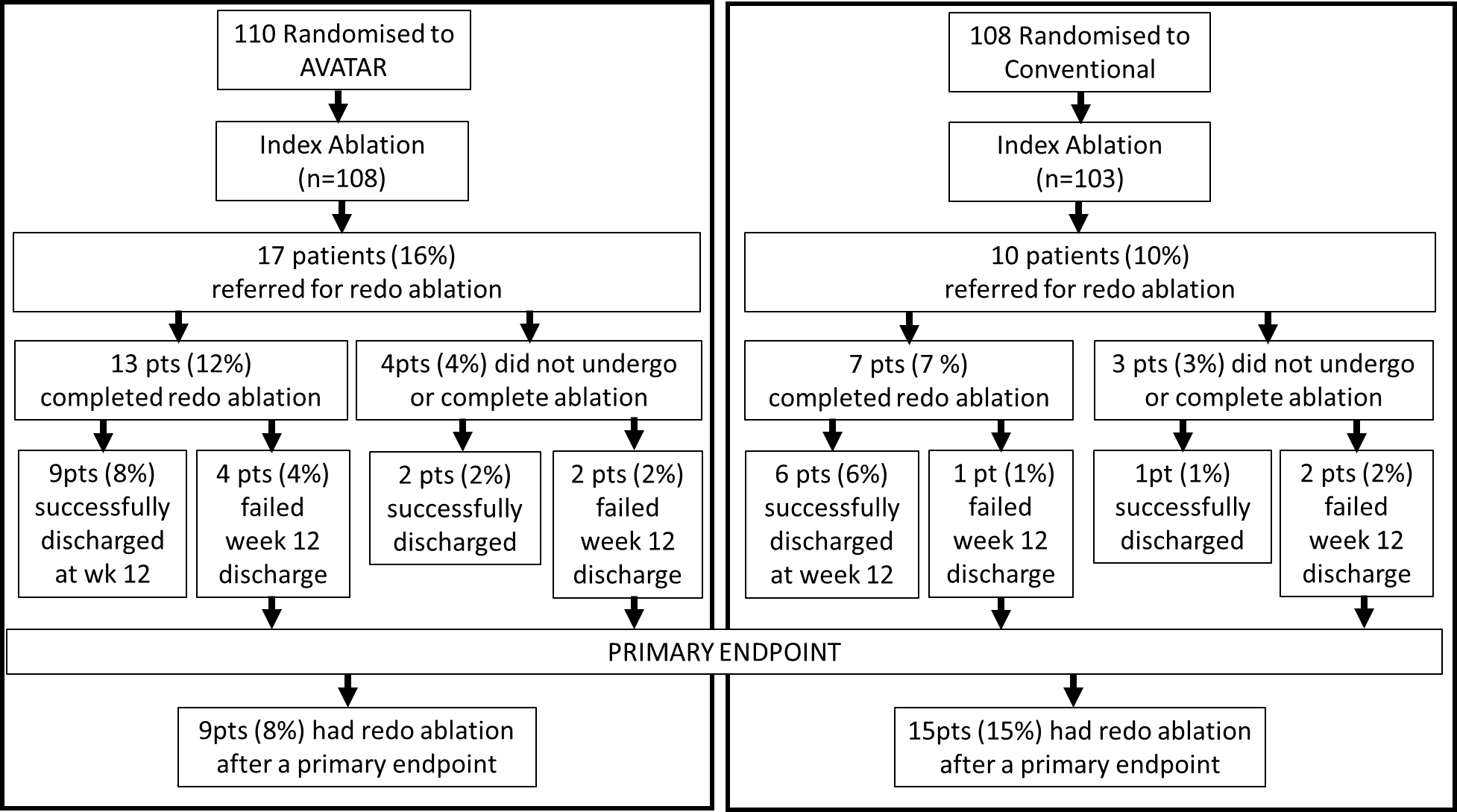
**

**APPENDIX FIGURE 1: Repeat Procedures in Ablation Arms.** Patient flow charts for both AVATAR protocol ablation and conventional ablation arms showing number and percentage of patients that were referred for a redo ablation procedure following index ablation. Referrals are subsequently categorised based on whether they a) completed their referred redo procedure and b) were successfully discharged at their week 12 review. Following primary endpoint of hospital episode related to treatment for atrial arrhythmia, 9 (8%) patients subsequently had a redo procedure in the AVATAR arm, with 15 (15%) post endpoint redo procedures within the Conventional arm. All percentages are calculated based on patients who had undergone an index ablation.

**
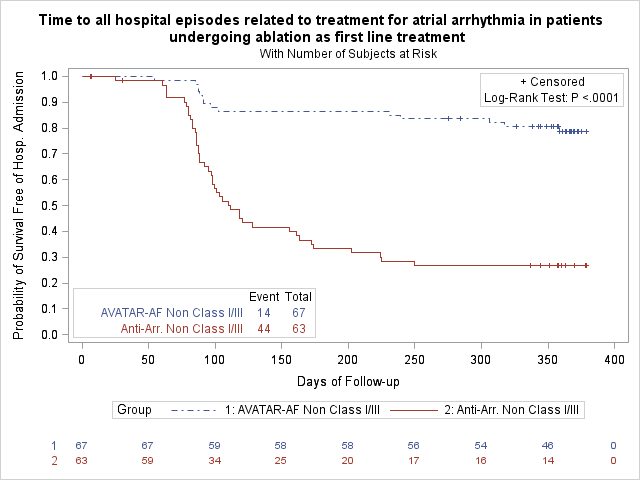
**

**APPENDIX FIGURE 2:** **Primary Endpoints in Primary Hypothesis: A post-hoc analysis of patients naïve to Class I/III anti-arrhythmic drugs.** The Kaplan-Meier curves comparing the sub-groups of patients naïve to Class I/III anti-arrythmic drugs within the AVATAR protocol ablation arm (n=67) against the drug therapy arm (n=63) for time to any hospital episode related to treatment for atrial arrhythmia. Significantly fewer patients from the AVATAR arm had reached the primary endpoint with 14 [21%] vs 44 [70%] patients having events within the two arms respectively (p<0.0001 by log-rank test).
